# Measuring the age-friendliness of cities in the Russian Federation: The translation, validation and application of the age-friendly cities and communities Questionnaire in the city of Kazan

**DOI:** 10.1016/j.heliyon.2024.e41100

**Published:** 2024-12-10

**Authors:** Liliya E. Ziganshina, Aizyara F. Garaeva, Liliya I. Talipova, Rustem N. Khairullin, Jeroen Dikken, Joost van Hoof

**Affiliations:** a Interregional Clinical Diagnostic Centre (ICDC), The Ministry of Health of the Republic of Tatarstan, 12A Karbysheva Street, 420101 Kazan, Russia; b Federal State Budgetary Educational Institution of Continuing Professional Education “Russian Medical Academy of Continuing Professional Education”, The Ministry of Health of the Russian Federation (RMANPO), 2/1, Barrikadnaya Street, 123995 Moscow, Russia; c Department of Pharmacology, Kazan State Medical University (KSMU), The Ministry of Health of the Russian Federation, 49 Butlerov Street, 420012 Kazan, Russia; d Department of General and Clinical Pharmacology, Peoples' Friendship University of Russia, Named After Patrice Lumumba (RUDN University, Named After Patrice Lumumba), The Ministry of Science and Higher Education of the Russian Federation, 6 Mikluho Maklaya Street, 117198, Moscow, Russia; e Research Group of Urban Ageing, Faculty of Social Work & Education, The Hague University of Applied Sciences, Johanna Westerdijkplein 75, 2521 EN Den Haag, the Netherlands; f Faculty of Health, Nutrition & Sport, The Hague University of Applied Sciences, Johanna Westerdijkplein 75, 2521 EN Den Haag, the Netherlands; g Department of Systems Research, Faculty of Spatial Management and Landscape Architecture, Wrocław University of Environmental and Life Sciences, Ul. Grunwaldzka 55, 50-357 Wrocław, Poland

**Keywords:** Scale, Quantitative, Evaluation, Assessment, Reliability, Validity

## Abstract

Numerous cities in the Russian Federation have joined the World Health Organization's (WHO) Global Network for Age-Friendly Cities and Communities since 2011. In order to do quantitative evaluations of the age-friendliness of cities, the Age-Friendly Cities and Communities Questionnaire (AFCCQ) was developed in the Netherlands. The purpose of this study was to translate and test the validity and reliability of the AFCCQ for use in the Russian Federation, and to study the views on the age-friendliness of the city of Kazan in the Republic of Tatarstan from an intergenerational perspective. Data were collected in a survey. In total, 208 people from various age cohorts met the inclusion criteria to assess the psychometric validity. Confirmatory factor analysis supported the structure with nine factors. Overall, the people in Kazan experience the age-friendliness of their city as positive. The youngest and oldest generations demonstrated the most positive scores. Only for the domain of respect and social inclusion results were reversed. The Russian language version of the AFCCQ proved a valid and reliable instrument to evaluate age-friendliness of cities and communities in Russia. Data gathered through the instrument can be used as input for planning, implementing and further monitoring of age-friendly initiatives in the country.

## 1 Introduction

The Russian Federation is one of the countries with a rapidly ageing population, which is partly caused by the decline in fertility rates, and has seen the emergence of various policy initiatives for health ageing and demographic development [[1], [2], [3], [4], [5], [6], [7]]. The general health of older Russians, their lifestyle and socio-economic status all have an impact on how they grow old and the level of independence they enjoy [[8], [9], [10]].

In order to improve the quality of life of older people and make societies more age-inclusive, over 1700 cities and communities all over the world have joined the World Health Organization's (WHO) Global Network for Age-Friendly Cities and Communities (GNAFCC), which is one of the most visible outputs of the organisation that emerged after the publication of the *Global Age-friendly Cities: A Guide* [11]. This guide was developed based on research with older people and other stakeholders in 33 cities worldwide, based on the Vancouver Protocol [12]. Among these 33 cities were two cities in the Russian Federation, namely the capital city of Moscow and Tuymazy in the Republic of Bashkortostan. During the validation research, which led to the publication of the global guide [11], it was shown that older Russians were rather satisfied with their public transportation options. Also, older Russians reported economic exclusion, they felt excluded from society due to low incomes and limited pensions. Back in 2007 many people found it too costly to purchase a computer, which excluded them from certain sources of news and information and limited them to only the radio, television and newspapers.

**Fig. 1:**
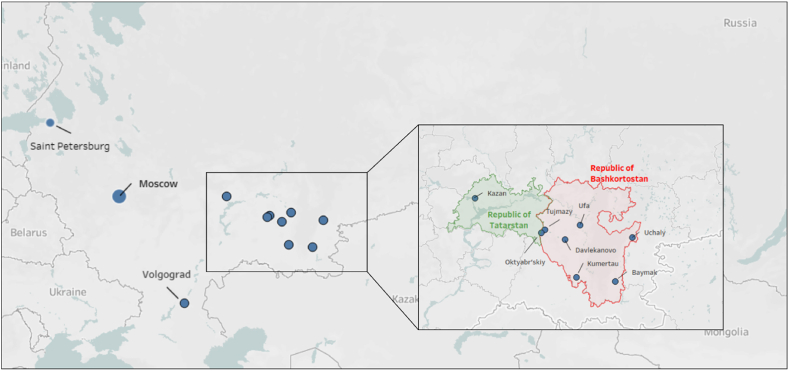
Map of the Russian Federation depicting the Russian members of the Global Network for Age-Friendly Cities and Communities and the boundaries of the Republics of Tatarstan and Bashkortostan. Transliteration of Russian place names according to the software used. Courtesy of Grzegorz Chrobak.

After the establishment of the GNAFCC in 2010, the following places in the Republic of Bashkortostan (in the Russian Federation) joined the network with the aim of stimulating them to become more age-friendly; Ufa (2011), Tuymazy (2011), Baymak (2011), Uchaly (2011), Oktyabrskiy (2011), Davlekanovo (2013), and Kumertau (2018) (see Fig. 1). Additionally, the city of Volgograd in the Southern Federal District joined the global network in 2012, as well as the second largest city in the Russian Federation, St. Petersburg in November 2024 [[89], [90]]. These cities and communities have committed themselves to propagating the principles of age-friendliness and to the 5-year Cycle of Continuous Improvement, which starts after signing a commitment letter. This encompasses a baseline assessment, making a strategy and action plan, and doing a formal evaluation. Ideally, older people are actively involved in the decision-making process as part of this cycle.

Evidence from Russia on age-friendly cities is very limited and dispersed [13]. conducted an exploratory study in the city of Kazan in the Republic of Tatarstan (Fig. 1) in particular, on the domain of community support and health services of the WHO model on age-friendly cities. A survey was conducted on the health information needs and the challenges that people face to improve their health and longevity. One of the outcomes pointed to a low level of awareness of ageism, with an overall positive assessment of the age-friendliness of Kazan [14]. described the development of age-friendly programmes in the city of Tuymazy. Among the plethora of strategic approaches undertaken, such as building on culture, and establishing partnerships with local government and other organizations in the area, there was specific attention on the language barrier as most of the available documentation was written only in English.

At the same time there is a recent body of literature in the domain of healthcare, such as studies on quality of life [15,16] prevention [17], and the relationship between food insecurity and depression among older adults [18], as well as other age-friendly parameters, namely those of social participation [19], the integration of healthcare, active ageing and social services [20,21], and the impact of social networks on cognitive function [22]. Others have researched the value attitudes of Russians towards older people [23], long-term care reform for dignified ageing [24], and the professional training of older citizens [25].

Prokofyeva et al. [26] published on the creation of health-oriented city spaces in a broader context than that of age-friendliness as a method to manage mainly environmental and behavioural health risks for the wider population. In their study, the scholars focused on a system of indicators that can be used to assess the level of development of health-oriented urban environments. Their indicators concern health-preserving as well as health-improving components [26]. Another study by Grigoryeva and Petukhova [27] provide a theoretical approach to the notions of ageing-in-place and the potential for this concept in Russia. Others have focused on active ageing in Russia and the application of the Active Ageing Index in the country [[28], [29], [30]]. There is also a body of knowledge concerning the use of modern communication technologies by older Russian citizens, for instance, concerning self-employment, the digital divide in society and digital health [[31], [32], [33], [34]].

The website of the WHO's Global Network [WHO, 2024 ab] describes the efforts of the Russian cities and communities and states that the member cities should focus on improving the accessibility of the city environment for older people in the widest possible fashion: to create favourable conditions for active and healthy ageing, to create places for social participation and activities including temporary care centres, and to develop a volunteering movement for older people. To date, it is unknown if these initiatives have been evaluated.

In general, the majority of members of the global network resort to qualitative methods for the formal evaluations and assessments [[35], [36], [37], [38], [39]]. Despite the rich outcomes of research using qualitative methods, there has been a strong need for a systematic quantitative method to measure the age-friendliness of cities and communities alike [35,[40], [41], [42], [43]]. In 2020, a validated instrument was developed in the Netherlands specifically for this purpose [35,38,44]. This 23-item Age-Friendly Cities and Communities Questionnaire (AFCCQ) [35] was later also validated for use in Turkey by Özer et al. [45], in Japan by Yamada et al. [46], Romania by Ivan et al. [47], North Macedonia by Pavlovski et al. [48], Israel by Ayalon et al. [49], Poland by Perek-Białas et al. [50], and Australia by Wasserman et al. [91]. More countries are to follow in the effort to test its validity and reliability for use in a specific national context.

At present, there is no quantitative instrument to evaluate the age-friendliness of cities and communities in Russia from an intergenerational perspective, which, in turn, could help steer the direction in which age-friendly initiatives move. The age-friendly model was based on the notions of active ageing [11], p.72 and it follows a life course perspective. It is stated that an age-friendly city emphasizes enablement rather than disablement, and is friendly for all ages and not just for older persons. This life course approach includes all ages within the process of promoting active ageing. It also embraces the value of intergenerational solidarity and fosters solidarity between generations and within communities, and facilities activities that bring together people of all ages [11]. It is even postulated that though a network of trusted relatives, friends, neighbours and service providers, older people should remain integrated within the community. Also, economic, linguistic or cultural barriers experienced by many older people should be minimised [11].

Therefore, this study tests the validity and reliability of a Russian version of the AFCCQ and presents the initial age-friendly results of the City of Kazan from the perspective of multiple generations. This study would add a Russian language version of the AFCCQ to the spectrum, which is of particular relevance given that Russian is one of the six official working languages of the United Nations.

## 2 Methodology

### 2.1 Setting

The city of Kazan, the capital city of the Republic of Tatarstan, was the setting of this validation study. Tatarstan has close cultural, linguistic, and ethnic ties with Bashkortostan, a neighbouring region with many cities active in the Global Network for Age-Friendly Cities and Communities. The city of Tuymazy was one of the 33 original cities taking place in the research leading to the publication of the global guide [11]. Tatars and Bashkorts are very close in all these dimensions, sharing the same language, traditions and history, with high proportions of ethnically mixed persons in the population.

Tatarstan is the most actively developing region of the Russian Federation. Tatarstan's population is 3.9 million people, which over the last five years has experienced a growth of over 0.5 %. The territory of Tatarstan is 67,836 square kilometres in surface area. It is one of the most densely populated regions of the Russian Federation (59 persons/km^2^), whilst the population density of Bashkortostan is about half (28 persons/km^2^) [51]. Tatarstan has experienced an increase in the ageing population, among men and women equally, in the cities and the countryside [52,53]. Kazan, the capital of Tatarstan, has a history dating back more than a millennium. It is situated on the left bank of the Volga River. The city has a population of 1,314,685 people as of 1 January 2023, and is the fifth largest in the Russian Federation [92]. Kazan is considered to be a comfortable city for its citizens in their everyday life, offering a safe and age-friendly urban environment [54]. In 2016, Tatarstan officially transitioned to a high level of old age in terms of its demographic structure: people aged 60 years and over represent more than 18 % of its population [52]. This study is the second one performed in Tatarstan that explores the concept of healthy ageing. The first study addressed the challenges of ageing in Tatarstan and explored the concept of the evidence-based approach for healthy ageing [13] using a mix of quantitative and qualitative methods.

The Demographic Yearbook of Russia [55], p. 31 shows that the people in the Russian Federation nowadays live longer. Looking at the graphs of the age and gender structure of population from 1959 to 2020, according to official censuses (1959; 1989; 2002; 2010 and 2020), we see that more and more people are living longer. This is reflected in the mean age of Russian population, which becomes higher, raising from 37.4 in 1990 to 43.0 in 2023. With a mean age of 40.6 years of urban populations in 2023, it makes the population of the Russian Federation relatively old. In 2020, the Russian Federation ranked fifth in terms of countries with the largest number of older adults, with an estimated 21.42 million people aged 65 years and over (14.6 % of the total population) [56]. Similar patterns of population ageing can be observed in both Tatarstan and Bashkortostan, following the national demographic report on well-being of the regions of the Russian Federation [93], with much higher numbers of older women than men; often twice as many.

### 2.2 Recruitment of participants

For this study, we follow the methodological steps taken by Yamada et al. [46], who also studied an intergenerational sample from Japan for the successful validation of the AFCCQ in their particular country. Cities that want to be age-friendly often focus on older age only. But in reality, becoming age-friendly is a phenomenon that transcends the needs of one particular generation and should be seen as an intergenerational challenge. Therefore, we have taken a transgenerational approach [57] as a route for future age-friendly strategies and long-term planning. In this concept, solidarity and the transmission of knowledge and practices between the generations is stimulated [57].

Therefore, this study made use of a sample that spans across the age-cohorts or generations (Table 1) asking both older people and their formal and informal carers as the spokespersons for older adults; in short, people who are either older adults or have a clear relationship to the topic of aged care and age-friendly environments.

**Table 1:** Demographics of participants Kazan, Russia (total = 293).

**Sex**	
Male	52 (17.7 %)
Female	241 (82.3 %)
**Age,** Mean (SD)	45.4 (16.99)
Age 20-29	71 (24.2 %)
Age 30-39	49 (16.7 %)
Age 40-49	48 (16.4 %)
Age 50-59	52 (17.7 %)
Age 60-69	45 (15.4 %)
Age 70+	28 (9.6 %)
**Educational level**	
ISCED 0-2	1 (0.3 %)
ISCED 3-4	211 (72.0 %)
ISCED 5-6	1 (0.3 %)
ISCED 7-8	80 (27.3 %)
**Income group**	
Low (1–20 thousand rubles per month)	54 (18.4 %)
Medium (21–90 thousand rubles per month)	231 (78.8 %)
High (91–150+ thousand rubles per month)	7 (2.4 %)
*Missing values*	1 (0.3 %)
**Type of dwelling**	
Owner-occupant	250 (85.3 %)
Private rent	33 (11.3 %)
Other (friend, municipal owner)	10 (3.4 %)
**Living together with a spouse or partner**	256 (87.4 %)
**Receiving care**	20 (6.8 %)
**Living with one or more chronic conditions**	144 (49.1 %)
*Missing values*	9 (3.1)
**Using a mobility aid**	0 (0 %)
**Self-rated quality of life** Mean (SD) (grade very 1 low – 10 very high)	7.91 (1.72)

The aim was to recruit at least 200 participants as per protocol for the validation of the Russian version of the AFCCQ based on structural equation modelling. A common rule of thumb is that the sample size should be 3 to 20 times the number of variables (i.e., items) in the model, and minimum sample sizes are often suggested to be 100 participants or over [58]. The analysis was based on only complete cases for the psychometric validation, which means that data from 208 participants were used for validation (see paragraph 2.6). This amounts to a ratio of 9:1 (nine persons for every item), well above the minimum of 100 participants. Moreover, the sample size did not affect the performance of analysis, as even smaller sample sizes have been shown to be sufficient [59,60].

Data collection took place between October and December 2023. We used online Google forms and involved the Cochrane Russia network to distribute the survey link and to invite people to participate. This approach yielded 269 respondents, only 14 of whom were aged 60 years and over.

In order to enroll a larger number of older participants, we additionally surveyed the older patients of the Interregional Clinical Diagnostic Centre (ICDC) of Kazan and their carers, as well as staff members taking care of older patients, providing them with paper survey forms and interviewing them face to face. This yielded 116 additional respondents.

In total, we recruited 385 participants. Of these 385 participants, 85 demonstrated missing values not at random (concerning the domain of Communication and Information) and were, therefore, excluded. Moreover, some people living as far away as Moscow (n = 7) were also excluded from further analyses. This led to the inclusion of a total of 293 people for the analyses (Table 1).

### 2.3 Ethics

This study obtained ethics approval from the Ethics Committee of the ICDC, numbered 133 dated 6 July 2023. All participants taking part in the online survey were informed that consent to participate in the study and publish their data would be assumed on completion and submission of the survey. All the participants from the ICDC provided written informed consent to participate in the study and for their data to be published.

### 2.4 Instruments

The data were collected online by the researchers using the "Demographic Information Form" and the "Age-Friendly Cities and Communities Questionnaire (AFCCQ)". The Demographic Information Form was created by the researchers and consists of 15 questions that help determine the socio-demographic characteristics of the people participating in this validation study (Table 1). The AFCCQ was developed by Dikken et al. [35] to evaluate the concept of age-friendliness. The scale consists of 23 items and nine domains and can be found in Dikken et al. [35]. The scale is answered using a 5-point Likert-type scale, with higher scores indicating a positive attitude towards the age-friendliness.

### 2.5 Research stages

The validation phase comprised a total of four distinct stages. The first stage concerned the language validity. The original Dutch AFCCQ, also available in British English, was translated into Russian by Google translate instrument, following the recommendations by Ziganshina et al. [61] and by two language experts both separately and independently, and then all three versions were reviewed by the team of authors. Only a few expressions that were translated slightly differently, were equalised. Hereafter, a single form was created that was re-reviewed by two Russian language experts. Minor corrections were made based on the assessment of the suitability of the scale items, the validity of the Russian translation, as well as the fit with the local culture. There items of the scale were then collected on a single form and translated back to into English by a foreign language expert. This translated form was then checked against the original scale, and it was concluded that the Russian end product was similar to original one written in English. In stage two, the instructions, items and response format clarity were assessed by six older people (face validity) and ten experts (content validity) in order to validate both the language and culture adaptation of the AFCCQ-RU using the method described by Lynn [62]. After this step, a confirmatory factor analysis (CFA) was carried out to test the construct validity using [35] original model. Stage four consisted of the testing of the composite reliability, which was followed by the assessment of the outcomes for the city of Kazan.

### 2.6 Translation, adaptation and validation

In order to assess cultural adaptation and qualitative validity of the AFCCQ-RU, the Item-Content Validity Index (I-CVI) is calculated as the percentage of agreement between the opinions of a minimum of five and a maximum of ten experts [[62], [94]]. For determining the I-CVI, experts are expected to respond each item with "not suitable (1)", "the item needs to be adjusted (2)", "appropriate but requires small changes (3)", or "very appropriate (4)". When using this method, the number of experts who marked (4) or (3) is divided by the total number of experts to obtain the I-CVI for each item. If the result of the analysis is greater than 0.78, it states that there is sufficient agreement in terms of item relevance among the raters, and that items with lower I-CVI may be taken out. Only if both older people and academic experts did not find an item to be relevant (both groups scored I-CVI lower than 0.78) an item was excluded.

For Confirmatory Factor Analysis (CFA), only cases having no missing values were included in the analysis, n = 208 (listwise deletion was applied). Then the variance to unity was set to allow factors to co-vary. To evaluate the fit with Dikken et al.’s [35] original model various fit indices were used. The normed χ^2^ was considered. This fit index is less sensitive to sample size than the χ^2^, with values up to 5 to evaluate model fit to be adequate [63]. The Comparative Fit Index (CFI) and Tucker Lewis Index (TLI) were used, which should be at least 0.9 [64]. The Root-Mean Squared Residual (SRMR) was also used (this index should be less than 0.08) [65] as well as the Root-Mean Square Error of Approximation (RMSEA) (this index should be less than 0.08 for a moderate fit) [66]. Finally, the internal consistency of the model was evaluated using composite reliability. The internal consistency should be over 0.70 [67]. The confirmatory factor analyses were conducted in IBM SPSS Amos version 28.0.

### 2.7 Analysis of the preliminary age-friendliness of the city of Kazan

A total 293 people were included in this study and 208 of this sample did not have any missing values on AFCCQ-RU items. After the validity of the AFCCQ-RU was determined, pairwise deletion was used for the 85 persons having (several) items missing at random, helping to maximise all data available for reporting results of the city of Kazan by an analysis-by-analysis basis [68]. These analyses were done using SPSS version 29.0.

## 3 Results

### 3.1 AFCCQ-RU translation, adaptation and initial validation

The process of forward translation was conducted by an independent, professional translator and the Google translate engine as described in Ziganshina et al. [61] The final wording was then decided upon through discussion and reaching consensus. Some small changes were made for a consistent use of the Russian language. The result of the process of back translation was linguistically very close to the original version of the AFCCQ. No further changes were necessary on the final version of the back translation, which was concluded based on consensus in the Russian team. The final AFCCQ-RU can be found in Appendix 1.

A total of 6 older persons (face validity), 4 females and 2 males aged 66–81, and 10 academic experts, 4 females and 6 males, specialised in the field of gerontology and/or ageing in urban contexts (content validity) scored the AFCCQ-RU items on relevance and readability (Table 2). None of them had comments or suggestions to improve the readability. Some items were considered to be irrelevant for the Russian context by older people or experts. None of the items were considered irrelevant by both groups and therefore all items remained for further analysis.

**Table 2:** Face and Content validity on AFCCQ-RU items. See Dikken et al. [35] for the full text descriptions of each of the items.

Domain and item	Face validity I-CVI (n = 6)	Content validity I-CVI (n = 10)
**Housing**		
Q1	1.0	1.0
Q2	1.0	0.3
**Social participation**		
Q3	1.0	0.0
Q4	1.0	1.0
Q5	1.0	1.0
Q6	1.0	1.0
**Respect and social inclusion**		
Q7	0.17	1.0
Q8	0.17	1.0
**Civic participation and employment**		
Q9	0.83	1.0
Q10	1.0	1.0
**Communication and information**		
Q11	1.0	0.0
Q12	1.0	0.0
**Community support and health services**		
Q13	0.83	1.0
Q14	1.0	1.0
Q15	1.0	0.9
Q16	1.0	0.9
Q17	1.0	0.9
**Outdoor spaces and buildings**		
Q18	0.83	1.0
Q19	0.83	1.0
**Transportation**		
Q20	1.0	1.0
Q21	1.0	1.0
**Financial situation**		
Q22	1.0	1.0
Q23	1.0	1.0

**Fig. 2:**
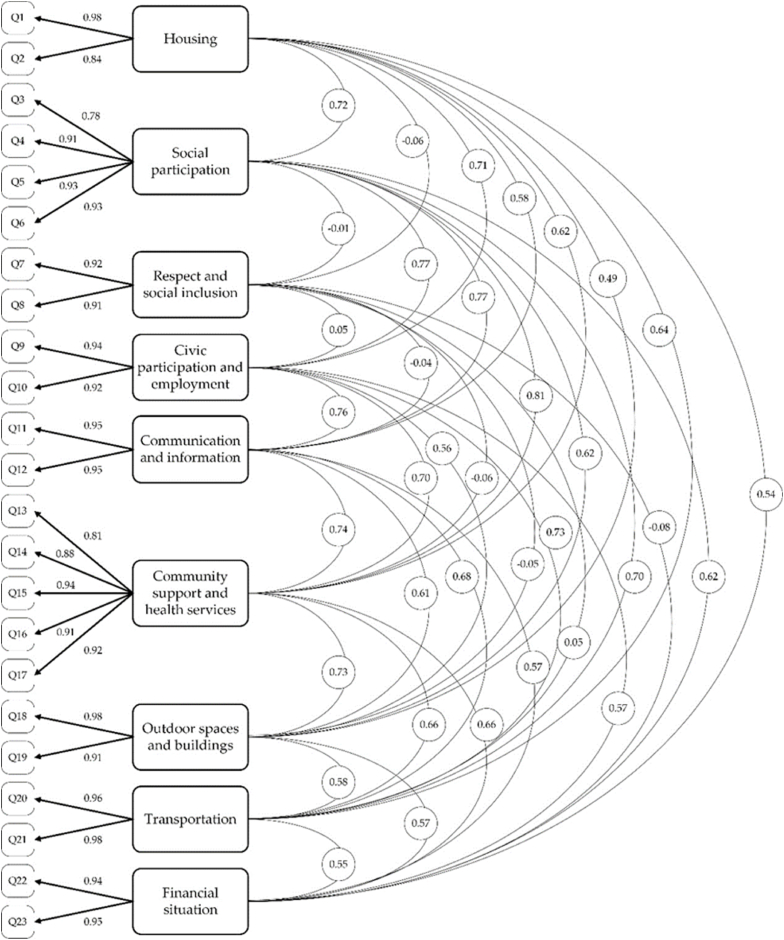
PATH diagram regarding the factor structure of the AFCCQ-RU.

The CFA model showed that the original factor model of the AFCCQ had a good fit with the data (Table 3). The normed χ^2^ was 2.651, which represents an adequate fit. Values of the CFI and TLI were 0.956 and 0.943 respectively. Both values are well above the 0.9 threshold [64]. The RMSEA was 0.077 and, therefore, adequate [66]. The robust SRMR was 0.0432, which is also below 0.08 [65]. The estimated covariance paths between the factors were below 0.85, which is an indication of sufficient discriminant validity. The final model is shown in Fig. 2.

**Table 3:** Fit of data from Russia with the original model as described by Dikken et al. [35].

Model	χ^2^*(DF)*	Normed χ^2^	Comparative Fit Index (CFI)	Tucker Lewis Index (TLI)	Root-Mean Squared Residual (SRMR)	Root-Mean Square Error of Approximation (RMSEA) 95 % *(90 % CI)*
	436.5 *(195)*^a^	2.651	0.956	0.943	0.0432	0.077 *(.068–.087)*

a sig level ≤0.001.

The final confirmatory factor analysis led to the emergence of model, of which its internal consistency was successfully tested through the composite reliability, which scores exceeding 0.70 (Table 4).

**Table 4:** Reliability per domain of the AFCCQ-RU.

Domains 1-5	Housing	Social participation	Respect and social inclusion	Civic participation and employment	Communication and information
**Composite Reliability**	0.909	0.938	0.912	0.927	0.952

Domains 6–9	Community support and health services	Outdoor spaces and buildings	Transportation	Financial situation	

**Composite Reliability**	0.952	0.943	0.971	0.948	

### 3.2 Preliminary results of the age-friendliness of Kazan

Overall, the city of Kazan has a satisfactory score regarding the age-friendliness of the city based on the outcomes of the AFCCQ. The highest scores were found for three domains, namely (1) Social participation; (2) Respect and social inclusion; and (3) Community support and health services. The domains of (4) Financial situation, and (5) Outdoor spaces and buildings received the lowest scores from the participants (Table 5).

**Table 5:** AFCCQ-RU results for the city of Kazan.

Total (n = 293)	Housing (n = 284)	Social participation (n = 264)	Respect and social inclusion (n = 285)	Civic participation and employment (n = 290)
14.93 ± 22.20 (++) *Missing:85*	2.36 ± 2.17 (++) *Missing:9*	3.18 ± 4.68 (++) *Missing:29*	2.91 ± 2.04 (+++) *Missing:8*	2.37 ± 2.22 (+++) *Missing:3*

Communication and information (n = 233)	Community support and health services (n = 291)	Outdoor spaces and buildings (n = 288)	Transportation (n = 291)	Financial situation (n = 292)

1.44 ± 2.58 (++) *Missing:60*	2.49 ± 6.19 (+) *Missing:2*	0.62 ± 2.64 (+) *Missing:5*	2.20 ± 2.50 (+++) *Missing:2*	0.36 ± 2.53 (+) *Missing:1*

**Fig. 3:**
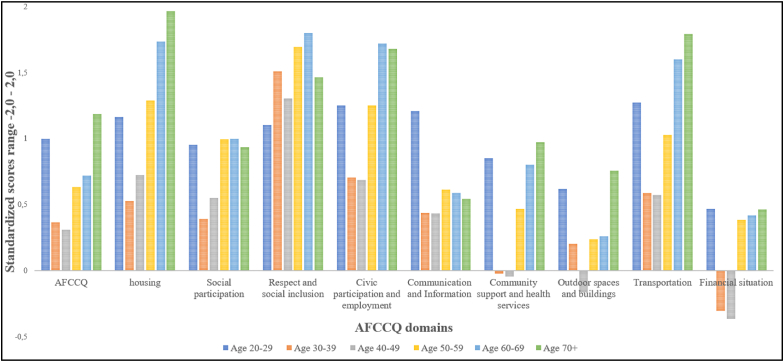
Standardised AFCCQ scores per domain and age cohort.

In Fig. 3, differences between the scores of age cohorts for the domains of the AFCCQ are presented. For the total AFCCQ, the youngest (20–29 years old) and oldest (70 years and over) are the most positive towards the age-friendliness of the city, leading in a prominent U-shape, in particular towards the domains of Housing, Outdoor spaces and buildings, Civic participation and employment, Community support and health services, Transportation and Financial situation. Remarkable is the score on the domain of Respect and social inclusion, where the youngest and oldest cohorts score the negative, meaning they experience the discrimination towards old age in Kazan (showing a reversed U-Shape). Regarding the domain of Communication and information, the difference between the youngest cohort and all other cohorts is the largest, meaning they believe the city of Kazan communicates adequately with older people even though the oldest older people (cohort aged 70+) are less positive. Overall, the respondents from the age group 30–49 are the most sceptical (negative) about the age-friendliness of Kazan. For numeric scores for the AFCCQ domains per age cohort, see Table 6.

**Table 6:**
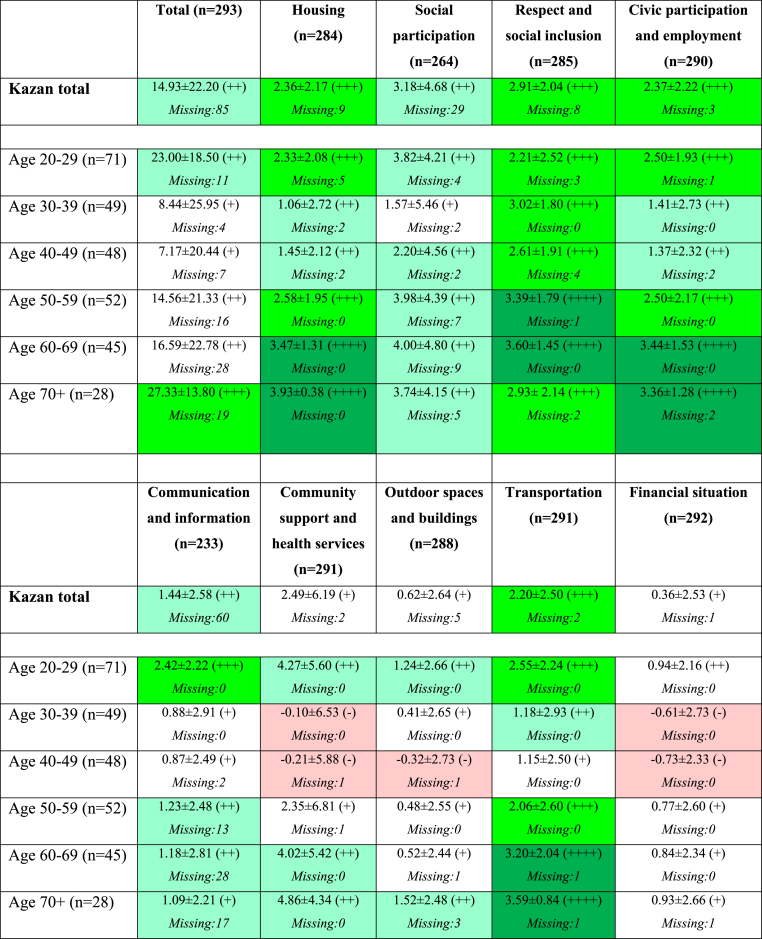
AFCCQ scores for Kazan and for different age groups.

The presentation of the scores in this table follows the method presented in Dikken et al. [35].

## 4 Discussion and implications

The Russian language version of the AFCCQ proved a valid and reliable measurement tool to evaluate the age-friendliness of the city of Kazan in the Russian Federation. The process of validation resulted in a valid and psychometrically sound instrument, which was named the Age-Friendly Cities and Communities Questionnaire - Russia [AFCCQ-RU]. This means that the AFCCQ is now available in the first of the six working languages of the United Nations and the World Health Organization, and that age-friendliness can now be examined and assessed among Russian speaking communities. The new AFCCQ-RU meets the recommendations for the evaluation of age-friendly city and community initiatives postulated by Buckner et al. [69]. The AFCCQ-RU may be used to collect baseline data from Russian cities, and add to the knowledge base concerning key characteristics of age-friendly cities and communities [[70], [71], [72]].

Considering the large number of cities in the Russian Federation that have become members of the WHO's Global Network for Age-Friendly Cities and Communities since 2011 and having the AFCCQ available in the largest of the Slavic languages for use in the largest country on earth, means that many communities can potentially benefit from the instrument. Moreover, as the validation took place in the multi-ethnic city of Kazan, the results provide a further opportunity for expansion of the applicability of the scale. Together with a recent validation in Turkish by Özer et al. [45], which led to the Turkish version of the AFCCQ, the two versions of the AFCCQ may be used to further adapt the tool for use in geographical areas in the Russian Federation where Turkic-speaking minorities live, as well as in Central Asian countries such as Kazakhstan, Turkmenistan, Uzbekistan and Kyrgyzstan, and in Azerbaijan, where the Turkic languages are also spoken. As the AFCCQ proved to be a valid instrument in Kazan, with its ethnically, religiously and culturally diverse population, there is a sound basis to assume that the instrument may be validated successfully in other Central Asian cities and communities, too.

When zooming in on the results from Kazan we see that overall, the results are positive, with the highest scores found for Respect and social inclusion, Civic participation and employment, and Transportation. The lowest scores, though still positive, were found for Community support and health services, Outdoor spaces and buildings, and Financial situation. People in Kazan overall experience the age-friendliness of the city as positive. The U-shape presented in results demonstrated that the youngest cohort is very capable in imagining how the city would be like for older people, more than other cohorts aged between 30 and 49 years, and often better than the cohort aged between 50 and 69 years. Their scores resembled the scores of older people the most. Overall, the youngest and oldest generations demonstrated the most positive scores, and only for the domain Respect and social inclusion were the results reversed. These findings may be because there is more mistrust between the generations due to the age difference, and the youngest generation more frequently hear their peers discriminate older people leading to the belief that older people experience a less age-friendly city [73]. In this study we asked younger people to answer the survey questions with their parents and grandparents in mind and with the additional consideration of themselves ageing, which could explain our findings.

Moreover, Respect and social inclusion are the two components that are most important both for the oldest citizens of Kazan and the youngest generation based on their beliefs, values and vulnerabilities. The oldest people have retired and have stopped being actively involved in economic development, and potentially might not feel needed and appreciated by the majority population of middle aged actively employed people. The same could be the case for the youngest people, who have not yet acquired secure job positions and/or are not yet included in roles matching their ambitions. However, to test these hypotheses we should extend our research project further, reaching out with the Questionnaire to many more people, involving people from all ages, other regions and municipalities of the Russian Federation and the Republic of Tatarstan, and potentially add a few open-ended qualitative questions.

One of the most striking features of the results from Kazan is the gap between Civic participation and employment, and Financial situation. This could be due to the pension politics and the welfare regime in Russia, the participation of older people in the labour market, and the limited purchase power of some older people [[74], [75], [76], [77], [78], [79]]. [80] have even explored the potential of the Silver Economy in the former Soviet Union, and in general, the ageing of the Russian population and its impact on the country's economy. This has also been studied in great detail by other Russian scholars [81,82]. Data collection using the AFCCQ-RU may further add to the knowledge base as it can be collected directly from the experience of older people themselves.

This study in Kazan was the first time that the AFCCQ was validated using respondents from multiple generations, proving that the AFCCQ is valid for use in the Russian Federation and could also be applied to other age groups. Overall, the validation procedure as undertaken in this study in Russia stresses the importance of transgenerational and intergenerational approaches in the study of ageing [57,83]. It also shows that in many domains of the AFCCQ and the age-friendly cities and communities’ model of the [11] people from various generation have the same opinions and beliefs.

Another strength of the present validation was the absence of missing data. Such absence leads to an increased validity of the results. The AFCCQ-RU can now be considered psychometrically valid. This also means that so-called imputation of data can be applied in any studies that are going to be conducted in the Russian Federation. One limitation is that we do not know exactly with what perspective people truly answered the AFCCQ questions, only from their own perspective (and thus their generation) or from the perspective of whether they believe the city is age-friendly for older people. Therefore, it is difficult to draw conclusions from these results on how age-friendly the city of Kazan is in practice, other than that the AFCCQ is valid for use by different generations. Further research is therefore sought with a larger sample of older adults in Russia (at least 200) and younger counterparts (at least 200 for each of the cohorts younger than 65 years of age) to fully validate the intergenerational perspective and to draw conclusions on the actual age-friendliness of the city of Kazan. The present study outcomes do, however, provide a positive foundation for such an endeavour, and together with the study by Yamada et al. [2024] from Japan, form a new approach for other countries to follow.

Research on the health promotion of the ageing population has been carried out primarily in the major cities of Moscow and Saint Petersburg, and the latter city has joined the Global Network in late 2024. Surveys and qualitative and quantitative assessments have been conducted since the 1990s [84,85]. The most substantial monitoring of ageing Muscovites was carried out in the framework of a longitudinal study (2011–2015) by the Centre for Monitoring Studies. Special emphasis was put on the needs of the ageing population and their change over time [86]. The quality of life and emotions of the older people as well as at the social and economic factors were studied. Many of the Russian research efforts are directed towards studying the information and training needs of older adults in the light of healthy ageing [87,88]. Nowadays many state-funded programmes of healthy ageing and active ageing are being implemented all over Russia, Tatarstan, and in Kazan. The programme of active ageing in Kazan this year is focused on family values [95]. This “Active Longevity project” is aimed at creating additional conditions for improving the quality and lifespan of older citizens by involving them in health, social and creative projects [96].

## 5 Conclusions

The newly validated 23-item AFCCQ-RU covers the eight original domains constituting the WHO's Age-Friendly Cities and Communities model, and an additional domain of the older person's financial situation. This is in line with findings from other countries where the validation took place. The AFCCQ-RU can be used in the Russian Federation to assess the age-friendliness of a city or community in a quantitative manner. It is foreseen that future research with the newly-validated AFCCQ-RU in Kazan and elsewhere in the Russian Federation will allow a better understanding of the needs of the ageing populations and inform future initiatives and programmes to be tailored precisely to the researched needs of the citizens of Russia and other Russian speaking countries. The data collected using the instrument can assist cities and communities in the governance of age-friendliness, following the 5-years Cycle of Continuous Improvement that members of the Global Network are requested to commit to by the WHO.

## CRediT authorship contribution statement

**Liliya E. Ziganshina:** Writing – review & editing, Writing – original draft, Project administration, Methodology, Investigation, Data curation, Conceptualization. **Aizyara F. Garaeva:** Writing – review & editing, Investigation, Data curation. **Liliya I. Talipova:** Writing – review & editing, Project administration, Investigation, Conceptualization. **Rustem N. Khairullin:** Writing – review & editing, Writing – original draft, Supervision. **Jeroen Dikken:** Writing – review & editing, Writing – original draft, Validation, Project administration, Methodology. **Joost van Hoof:** Writing – review & editing, Writing – original draft, Supervision, Funding acquisition.

## Ethics

This study obtained ethics approval from the Ethics Committee of the Interregional Clinical Diagnostic Centre, numbered 133 dated 6 July 2023. All participants taking part in the online survey were informed that consent to participate in the study and publish their data would be assumed on completion and submission of the survey. All the participants from the Interregional Clinical Diagnostic Centre provided written informed consent to participate in the study and for their data to be published.

## Data availability statement

Data will be made available on request.

## Funding

This publication was initiated through the participation of Liliya E. Ziganshina and Joost van Hoof in COST Action CA17117 “Towards an International Network for Evidence-based Research in Clinical Health Research (EVBRES)”, which is supported by COST (European Cooperation in Science and Technology). www.cost.euandhttps://evbres.eu/. The original dataset of the 2020 survey in the Netherlands was developed through funding from the Municipality of The Hague, grant number 10.13039/501100003245OCW/2020.1121.

## Declaration of competing interest

The authors declare the following financial interests/personal relationships which may be considered as potential competing interests: Joost van Hoof reports travel was provided by COST Action CA17117. Liliya E. Ziganshina reports travel was provided by COST Action CA17117. If there are other authors, they declare that they have no known competing financial interests or personal relationships that could have appeared to influence the work reported in this paper. This work has been conducted on a bilateral basis between scholars from the Russian Federation and the Netherlands in order to advance the knowledge in the field of age-friendly cities.

## Figures and Tables

**Table:** 

D1. Ваш пол?	Мужской	Женский	
D2. Год рождения?	Год рождения		
D3. В какой стране вы родились?	страна		
D4. Какое у Вас образование?	Уровень образования		
D5. В каком районе/городе вы живете? Ваш почтовый индекс?	Список соседей по городам (на выбор)		
D6. Как долго Вы живете в городе ∗указать город ….∗?	Живу на селе До 1 года 1–3 года Более 3-х лет		
D7. Вы живете в собственном доме, социальном жилье или частной арендованной собственности?	Собственный дом	Социальное жилье	Частная аренда
D8. Вы живете с кем-то или один?	Вместе с кем-либо	Один	
D9. Получаете ли вы уход на дому? (например, уборка, уход за собой)	Да	Нет	
D10. У вас есть хроническое заболевание или состояние? В7	Да	Нет	
D11. Вы пользуетесь ходунками или инвалидной коляской?	Да	Нет	
D12. Какую оценку вы хотели бы поставить качество своей жизни?	Оценка 1 - 10		

**Table:** 

**Часть 1: Жилье**						
1. Мой дом доступен для меня	1	2	3		4	5
2. Мой дом доступен для людей, которые приходят ко мне в гости	1	2	3		4	5
**Часть 2: Социальное участие**						
3. В моем районе достаточно возможностей познакомиться с людьми	1	2	3		4	5
4. Мероприятия и мероприятия организуются в доступных мне местах	1	2	3		4	5
5. Мне достаточно информации о мероприятиях, а также они подходят для меня	1	2	3		4	5
6. Я нахожу спектр событий и мероприятий достаточно разнообразным	1	2	3		4	5
**Часть 3:** Уважение и социальная интеграция						
7. Иногда я получаю раздражающие или негативные замечания из-за своего возраста	1	2	3		4	5
8. Иногда я сталкиваюсь с дискриминацией из-за своего возраста	1	2	3		4	5
**Часть 4:** Гражданское участие и занятость						
9. У меня достаточно возможностей для общения с молодым поколением	1	2	3		4	5
10. Я чувствую себя ценным членом общества	1	2	3		4	5
**Часть 5:** Связь и информация						
11. Печатная и цифровая информация от муниципалитета и других социальных учреждений легко читается с точки зрения шрифта и размера.	1	2	3		4	5
12. Печатная и цифровая информация от муниципалитета и других социальных учреждений написана понятным языком	1	2	3		4	5
**Часть 6: Поддержка сообщества и медицинские услуги**						
1. Мне достаточно заботы и благосостояния в моем городе	1		2	3	4	5
2. Когда я болен, я получаю уход и помощь, которые мне нужны	1		2	3	4	5
3. При необходимости я могу легко связаться со службами ухода и социального обеспечения по телефону и лично	1		2	3	4	5
4. У меня достаточно информации о службах ухода и социального обеспечения в моем районе	1		2	3	4	5
5. Работники по уходу и социальному обеспечению в моем районе достаточно уважительны	1		2	3	4	5
**Часть 7:Открытые пространства и здания**						
1. Мой район достаточно доступен для инвалидных колясок или инвалидных колясок.	1		2	3	4	5
2. Магазины в моем районе достаточно доступны для ходунков или инвалидной коляски.	1		2	3	4	5
**Часть 8: Транспорт**						
1. Я могу легко сесть на автобус или трамвай в моем районе	1		2	3	4	5
2. До автобусных и трамвайных остановок в моем районе легко добраться и ими легко пользоваться.	1		2	3	4	5
**Часть 9: Финансовое положение**						
1. Мой доход достаточен для покрытия моих основных потребностей без каких-либо проблем	1		2	3	4	5
2. Я хорошо живу на свой доход	1		2	3	4	5

## Appendix 1 Final version Translated AFCCQ-RU

Опросник городов и сообществ, дружественных к пожилым людям (AFCCQ) - Демография.

### **A.** Опросник городов и сообществ, благоприятных для пожилых людей

Пожалуйста, ответьте на вопрос по 5-балльной шкале Лайкерта.

1 Полностью не согласен
2 Не согласен
3 Нейтральный
4 Согласен
5 Полностью согласен

## References

[1] Golubeva E., Emelyanova A. (2021). Policy initiatives on healthy ageing in Russia from 2010-2020. Eur. J. Ment. Health.

[2] Dobrokhleb V.G. (2021). When society ages. Herald Russ. Acad. Sci..

[3] Fomicheva T.V. (2021). The dynamics of life-span of citizen of the Russian Federation: the sociological aspect. Problemy sotsial'noi gigieny, zdravookhraneniia i istorii meditsiny.

[4] Vorobyova O.D., Topilin A.V., Nioradze G.V., Khrolenko T.S. (2022). The demographic aging of population: regional trends in Russia. Problemy sotsial'noi gigieny, zdravookhraneniia i istorii meditsiny.

[5] Shcherbakova E.M. (2022). Population dynamics in Russia in the context of global trends. Stud. Russ. Econ. Dev..

[6] Strizhitskaya O., Petrash M., Golubitskaya D., Shchukin A., Engelgardt E. (2023). Futurization of aging: subjective beliefs and effects. Behav. Sci..

[7] Viktorovna-Blinova T., Gennadievna-Bylina S. (2021). Alternative scenarios of the demographic development of rural Russia: Analysis and forecast | [Escenarios alternativos para el desarrollo demográfico de la Rusia rural: Análisis y pronóstico]. Papeles Población.

[8] Larnyo E., Dai B., Nutakor J.A., Larnyo A., Appiah R. (2022). Examining the impact of socioeconomic status, demographic characteristics, lifestyle and other risk factors on adults' cognitive functioning in developing countries: an analysis of five selected WHO SAGE Wave 1 Countries. Int. J. Equity Health.

[9] Safarova G.L., Kipyatkova V.A., Safarova A.A. (2021). Effects of socio-economic factors on old-age mortality in Russia. Advances in gerontology = Uspekhi gerontologii.

[10] Gietel-Basten S., Mau V., Scherbov S., Shulgin S. (2021). The gender gap in reaching “old age” in the Russian federation: a regional approach. J. Aging Soc. Pol..

[11] World Health Organization (2007).

[12] World Health Organization (2007).

[13] Ziganshina L.E., Yudina E.V., Talipova L.I., Sharafutdinova G.N., Khairullin R.N. (2020). Smart and age-friendly cities in Russia: an exploratory study of attitudes, perceptions, quality of life and health information needs. Int. J. Environ. Res. Publ. Health.

[14] Minnigaleeva G.A., Caro F.G., Fitzgerald K.G. (2016). International Perspectives on Age-Friendly Cities.

[15] Rahman M.H.U., Srivastava S., Kumar P., Gupta D., Kaur V. (2022). Effect of disability on high quality of life among older adults in low and middle-income countries. Ageing Int..

[16] Shibalkov I.P., Nedospasova O.P., Pavlova I.A., Rozhdestvenskaia E.M. (2021). Satisfaction with the quality of life in the Russian regions in the context of realizing the resource potential of elderly people. Advances in gerontology = Uspekhi gerontologii.

[17] Anisimov V.N., Bordovskiy G.A., Finagentov A.V., Shabrov A.V. (2022). Innovative concept of aging prevention for modern Russia. Advances in gerontology = Uspekhi gerontologii.

[18] Smith L., Il Shin J., McDermott D., Grabovac I., Koyanagi A. (2021). Association between food insecurity and depression among older adults from low- and middle-income countries. Depress. Anxiety.

[19] Smith L., Shin J.I., López Sánchez G.F., Tully M.A., Koyanagi A. (2022). Social participation and mild cognitive impairment in low- and middle-income countries. Prev. Med..

[20] Prisiazhniuk D., Holavins A. (2023). Active ageing and social services: the paradox of empowerment in Russia. Eur. Asia Stud..

[21] Selezneva E.V., Sinyavskaya O.V., Gorvat E.S. (2022). Integration of medical and social services for the elderly in Russia: successes and barriers. [Интеграция медицинского и социального обслуживания пожилых в России: Успехи и барьеры]. Public Administration Issues.

[22] Nie Y., Richards M., Kubinova R., Bobak M., Ruiz M. (2021). Social networks and cognitive function in older adults: findings from the HAPIEE study. BMC Geriatr..

[23] Zaychikova I.V., Patsakula I.I., Khachikyan E.I. (2023). Value attitudes towards older people as social sustainability. Lecture Notes in Networks and Systems.

[24] Nizamova A., Zdravomyslova E. (2023). ‘Dignified ageing’: entrepreneurs of long-term care reform in Russia. Eur. Asia Stud..

[25] Rezer T.M. (2021). Methodology for the organisation of professional training of senior citizens: general concept. Методология организации профессионального обучения граждан старшего возраста: Общая концепция] Obrazovanie i Nauka.

[26] Prokofyeva A.V., Lebedeva-Nesevrya N.A. (2018). Creation of health-oriented city space as a way to manage population health risk | [Формирование здоровьеориентированного городского пространства как способ управления рисками здоровью населения]. Health Risk Analysis.

[27] Grigoryeva I.A., Petukhova I.S. (2022). Theoretical approach «Aging-in-place» and the possibility of its popularization in Russia. Advances in gerontology = Uspekhi gerontologii.

[28] Sinyavskaya O.V., Cherviakova A.A. (2022). Active aging in Russia during economic stagnation: what can we learn from the dynamics of the active agеing index?. [Активное долголетие в России в условиях экономической стагнации: что показывает динамика индекса активного долголетия?] Monitoring Obshchestvennogo Mneniya: Ekonomicheskie i Sotsial'nye Peremeny.

[29] Varlamova M., Sinyavskaya O. (2021). Active ageing index in Russia - identifying determinants for inequality. Journal of Population Ageing.

[30] Frolova E.A., Malanina V.A. (2021). Active ageing index in Siberian regions | [Индекс активного долголетия в регионах Сибири]. Economy of Regions.

[31] Vidiasovа L.A., Kuznetsova E.M., Grigoryeva I.A. (2022). Integration of the elderly into the information space: research case of Saint-Petersburg. Advances in gerontology = Uspekhi gerontologii.

[32] Kartuzova M. (2022). The digital-based self-employment of ageing population: the dream of one's life or monetization of one's network. [Электронна я самозанятость в старшем возрасте: Мечта д линою в жизнь и монетизация деловых связей] Zhurnal Issledovanii Sotsial'noi Politiki.

[33] Varlamova YuA. (2022). The intergenerational digital divide in Russia [?ежпоколенческий цифровой разрыв в ?оссии. Mir Rossii.

[34] Volkova O.A., Budarin S.S., Smirnova E.V., Elbek YuV. (2021). Experience of using telemedicine technologies in healthcare systems of foreign countries and the Russian Federation: systematic review. [Опыт использования телемедицинских технологий в системах здравоохранения зарубежных стран и Российской Федерации: систематический обзор] Farmakoekonomika.

[35] Dikken J., van den Hoven R.F.M., van Staalduinen W.H., Hulsebosch-Janssen L.M., van Hoof J. (2020). How older people experience the age-friendliness of their city: development of the Age-Friendly Cities and Communities Questionnaire. Int. J. Environ. Res. Publ. Health.

[36] van Hoof J., Dikken J., Buttiġieġ S.C., van den Hoven R.F.M., Kroon E., Marston H.R. (2020). Age-friendly cities in The Netherlands: an explorative study of facilitators and hindrances in the built environment and ageism in design. Indoor Built Environ..

[37] van Hoof J., Marston H.R., Kazak J.K., Buffel T. (2021). Ten questions concerning age-friendly cities & communities and the built environment. Build. Environ..

[38] van Hoof J., van den Hoven R.F.M., Hess M., van Staalduinen W.H., Hulsebosch-Janssen L.M.T., Dikken J. (2022). How older people experience the age-friendliness of The Hague: A quantitative study. Cities.

[39] Huisman M., Mysyuk Y. (2020). Older people's emotional connections with their physical urban environment. Cities & Health.

[40] Torku A., Chan A.P.C., Yung E.H.K. (2021). Age-friendly cities and communities: a review and future directions. Ageing Soc..

[41] Orpana H., Chawla M., Gallagher E., Escaravage E. (2016). Developing indicators for evaluation of age-friendly communities in Canada: process and results. Health Promotion and Chronic Disease Prevention in Canada.

[42] Garner I.W., Holland C.A. (2020). Age-friendliness of living environments from the older person's viewpoint: development of the Age-friendly Environment Assessment Tool. Age Ageing.

[43] Kim K., Buckley T., Burnette D., Kim S., Cho S. (2022). Measurement indicators of age-friendly communities: findings from the AARP age-friendly community survey. Gerontol..

[44] van Hoof J., van Staalduinen W.H., Dikken J. (2024). A multi-year quantitative study of the experienced age-friendliness in The Hague: A tale of four personas. Soc. Sci. Med..

[45] Özer Z., Turan G.B., Teke N. (2022). Age-friendly cities and communities questionnaire: a research on Turkish validity and reliability. Arch. Environ. Occup. Health.

[46] Yamada K., Murotani K., Mano M., Lim Y., Yoshimatsu J. (2023). Age-friendly approach is necessary to prevent depopulation: resident architectural designers and constructors' evaluation of the age-friendliness of Japanese municipalities. Int. J. Environ. Res. Public Health.

[47] Ivan L., Dikken J., van Hoof J. (2024). Unveiling the experienced age-friendliness of older people in bucharest: a comprehensive study using the validated Romanian age-friendly cities and communities questionnaire using cluster analysis. Habitat Int..

[48] Pavlovski D., Dikken J., Bajrami Ollogu E., van Hoof J. (2024). How older adults experience the age-friendliness of Skopje: results of the validation of the AFCCQ for use in North Macedonia and a representative survey. Heliyon.

[49] Ayalon L., Dikken J., van Hoof J. (2024). The age-friendly cities and communities questionnaire: a validation study of the Hebrew version in Israel. Heliyon.

[50] Perek-Białas J.M., Skórska P., Maj M., Kazak J.K., Dikken J., van Hoof J. (2024). The experienced age-friendliness in two Polish cities: an in-depth analysis of the views of older citizens. Habitat Int..

[51] Federal State Statistics Service (2024). Оценка численности постоянного населения на 1 января 2024 г. и в среднем за 2023 г. и компоненты её изменения. Федеральная служба государственной статистики. Дата обращения: 27 апреля.

[52] Fakhrutdinova E.V., Yagudin R.K., Rybkin L.I. (2016). Economic and Legal Issues.

[53] RIA RATING (2022). Rating of regions by demography. https://riarating.ru/infografika/20220404/630220607.html.

[54] Duma K.C., Duma K.S. (2023). On the Strategy of Socio-Economic Development of the Municipal Formation of Kazan until 2030.

[55] Rosstat2 (2023). Population of the Russian federation by municipalities. https://rosstat.gov.ru/compendium/document/13282.

[56] Population Reference Bureau (2020). Resource Library. Countries with the older populations in the world. https://www.prb.org/resources/countries-with-the-oldest-populations-in-the-world/.

[57] Marston H.R., Shore L., Stoops L., Turner R.S. (2022).

[58] Mundform D.J., Shaw D.G., Ke T.L. (2005). Minimum sample size recommendations for conducting factor analyses. Int. J. Test..

[59] Wolf E.J., Harrington K.M., Clark S.L., Miller M.W. (2013). Sample size requirements for structural equation models an evaluation of power, bias, and solution propriety. Educ. Psychol. Meas..

[60] Sideridis G., Simos P., Papanicolaou A., Fletcher J. (2014). Using structural equation modeling to assess functional connectivity in the brain: power and sample size considerations. Educ. Psychol. Meas..

[61] Ziganshina L.E., Yudina E.V., Gabdrakhmanov A.I., Ried J. (2021). Assessing human post-editing efforts to compare the performance of three machine translation engines for English to Russian translation of Cochrane plain language health information: results of a randomised comparison. Informatics.

[62] Lynn M.R. (1986). Determination and quantification of content validity. Nurs. Res..

[63] Schumacker R.E., Lomax R.G. (2004).

[64] Hu L.T., Bentler P.M., Hoyle R.H. (1995). Structural Equation Modelling: Concepts, Issues, and Applications.

[65] Hu L.T., Bentler P.M. (1999). Cutoff criteria for fit indexes in covariance structure analysis: conventional criteria versus new alternatives. Structural Equation Modelling: A Multidiscip. J..

[66] MacCallum R.C., Browne M.W., Sugawara H.M. (1996). Power analysis and determination of sample size for covariance structure modeling. Psychol. Methods.

[67] Hair J., Hult C., Ringle C.M., Sarstedt M. (2014).

[68] Peugh J.L., Enders C.K. (2004). Missing data in educational research: a review of reporting practices and suggestions for improvement. Rev. Educ. Res..

[69] Buckner S., Pope D., Mattocks C., Lafortune L., Dherani M., Bruce N. (2019). Developing Age-Friendly Cities: an evidence-based evaluation tool. Journal of Population Ageing.

[70] Steels S. (2015). Key characteristics of age-friendly cities and communities: a review. Cities.

[71] Lui C.W., Everingham J.A., Warburton J., Cuthill M., Bartlett H. (2009). What makes a community age-friendly: a review of international literature. Australas. J. Ageing.

[72] Plouffe L., Kalache A. (2010). Towards global age-friendly cities: determining urban features that promote active aging. J. Urban Health.

[73] van den Heuvel W.J. (2012). Discrimination against older people. Rev. Clin. Gerontol..

[74] Prisiazhniuk D., Sokhey S.W. (2023). Cracking the nest egg: comparing pension politics in post-communist Russia and Hungary. Soc. Pol. Soc..

[75] Cook L.J., Titterton M. (2023). Mapping shifts in Russian and European welfare polities: explaining policy responses to shared new social risks. Soc. Pol. Soc..

[76] Galkin K.A. (2021). Employment of older people and active agеing policies in Europe and Russia. Sotsiologicheskie Issled..

[77] Lyashok V.Yu, Varshavskaya E.Ya (2022). Interregional differentiation of the age of exit from the labour market in Russia. Economy of Regions.

[78] Chauhan S., Rahman M.H.U., Jaleel A., Patel R. (2022). Economic inequality in social cohesion among older adults in low and middle-income countries. Ageing Int..

[79] Vasilyeva E.V., Tyrsin A.N. (2021). Influence of employment incentives for seniors on the pension coverage in Russia. Smart Innovation, Systems and Technologies.

[80] Shestakova N.N., Djanelidze M.G., Skvortsova M.B. (2022). The silver Economy in the post-soviet space. Advances in Science, Technology and Innovation.

[81] Lukyanets A., Okhrimenko I., Egorova M. (2021). Population aging and its impact on the country's Economy. Soc. Sci. Q..

[82] Artamonov N.V., Kurbatskii A.N., Khalimov T.M. (2021). Relationship between economic development and population age structure in the Russian regions. [Взаимосвязь экономического развития и возрастной структуры населения регионов Российской Федерации] Terra Economicus.

[83] Ayalon L., Chasteen A., Diehl M., Levy B.R., Neupert S.D., Rothermund K., Tesch-Römer C., Wahl H.W. (2021). Aging in times of the COVID-19 Pandemic: avoiding ageism and fostering intergenerational solidarity. J. Gerontol.: Series B.

[84] Belokon O.V. (2005). Modern problems of the quality of life of the elderly in Russia (results of surveys). Adv. Gerontol..

[85] Shmeleva E.V. (2005). Elderly Petersburgers today: factors of quality of life. J. Sociol. Soc. Anthropol..

[86] Kornilova M.V. (2016). Sociological monitoring of the level and quality of life of elderly Muscovites: new methodological and organisational approaches. Sci. Result Ser. Sociol. Manag..

[87] Grokhotova E.V. (2018). Teaching the basics of computer literacy for people of the third age: problems and solutions. Bull. Sib. State Univ. Railw. Humanit. Res..

[88] Grigorieva S.A. (2018). Formation of conditions for social activity of older citizens. Soc. Humanit. Knowl..

[89] World Health Organization (2024). WHO global network for age-friendly cities and communities (arcgis.com). https://who.maps.arcgis.com/apps/instant/minimalist/index.html?appid=66799d4ec039487e8ef8367f0254a99a.

[90] World Health Organization (2024). https://extranet.who.int/agefriendlyworld/search-network/?_sft_countries=russian-federation.

[91] Wasserman R., Barrie H., Dikken J., van Hoof J., Soebarto V., Validating the Age-Friendly Cities and Communities Questionnaire in Australia: revealing five distinct groups of older people in Greater Adelaide, Habitat Int. (2025). (in press).

[92] Rosstat (2023). The demographic Yearbook of Russia. Statistical Handbook/D 31 Rosstat. - M., 2023 – 256 p. The Editorial Board: S.M. Okladnikov – Chairman of the Editorial Board S.Yu. Nikitina – Vice chairman of the Editorial Board E.M. Andreev, L.A. Vologirova, O.D. Vorobieva, M.B. Denisenko, A.E. Ivanova, V.A. Iontcev, A.S. Moruga, T.L. Kharkova, O.S. Chudinovskikh, V.G. Chumarina.

[93] Демографическое самочувствие регионов России. Национальный демографический доклад (2022).

[94] Polit D.F., Beck C.T., Owen S.V. (2007). Is the CVI an acceptable indicator of content validity? Appraisal and recommendations. Res. Nurs. Health.

[95] Ministry of Labour, Employment and Social Services of the Republic of Tatarstan (2022). В Казани реализуется программа «Активное долголетие»https://mtsz.tatarstan.ru/index.htm/news/2109498.htm. (Accessed 20 September 2024).

[96] Social service organization (ОРГАНИЗАЦИИ СОЦИАЛЬНОГО ОБСЛУЖИВАНИЯ НАСЕЛЕНИЯ) [2024] «Активное долголетие» [active longevity]. https://sobes.tatarstan.ru/aktivnoe-dolgoletie-6377569.htm.

